# Vibration-Based Damage Detection Using Finite Element Modeling and the Metaheuristic Particle Swarm Optimization Algorithm

**DOI:** 10.3390/s22145079

**Published:** 2022-07-06

**Authors:** Ilias Zacharakis, Dimitrios Giagopoulos

**Affiliations:** Department of Mechanical Engineering, University of Western Macedonia, Bakola and Sialvera, 50100 Kozani, Greece; izacharakis@uowm.gr

**Keywords:** damage detection, damage localization, vibration-based, model-based, FE model updating, metaheuristic algorithms

## Abstract

The continuous development of new materials and larger and/or more complex structures drives the need for the development of more robust, accurate, and sensitive Structural Health Monitoring (SHM) techniques. In the present work, a novel vibration-based damage-detection method that contributes into the SHM field is presented using Metaheuristic algorithms coupled with optimal Finite Element Models that can effectively localize damage. The proposed damage-detection framework can be applied in any kind of detailed structural FE model, while requiring only the output information of the dynamic response of the structure. It can effectively localize damage in a structure by highlighting not only the affected part of the structure but also the specific damaged area inside the part. First, the optimal FE model of the healthy structure is developed using appropriate FE model updating techniques and experimental vibration measurements, simulating the undamaged condition. Next, the main goal of the proposed method is to create a damaged FE model that approximates the dynamic response of the damaged structure. To achieve this, a parametric area is inserted into the FE model, changing stiffness and mass to simulate the effect of the physical damage. This area is controlled by the metaheuristic optimization algorithm, which is embedded in the proposed damage-detection framework. On this specific implementation of the framework, the Particle Swarm Optimization (PSO) algorithm is selected which has been used for a wide variety of optimization problems in the past. On the PSO’s search space, two parameters control the stiffness and mass of the damaged area while additional location parameters control the exact position of the damaged area through the FE model. For effective damage localization, the Transmittance Functions from acceleration measurements are used which have been shown to be sensitive to structural damage while requiring output-only information. Finally, with proper selection of the objective function, the error that arises from modeling a physical damage with a linear damaged FE model can be minimized, thus creating a more accurate prediction for the damaged location. The effectiveness of the proposed SHM method is demonstrated via two illustrative examples: a simulated small-scale model of a laboratory-tested vehicle-like structure and a real experimental CFRP composite beam structure. In order to check the robustness of the proposed method, two small damage scenarios are examined for each validation model and combined with random excitations.

## 1. Introduction

Over the last years, there has been an increasing rate of adoption of Structural Health Monitoring (SHM) systems due to the increasing development of new materials and larger and more complex structures. Moreover, this rate can be attributed to other factors as well, such as the cost of traditional maintenance, inspection, and monitoring procedures that are also prone to human error and are time-consuming. Preventive maintenance is vital for planning cost-effective solutions to preserve existing structures, to delay future deterioration, and to maintain and improve their functionality. A fault or damage on operating structures may cause sudden changes in their responses leading to potentially severe economic and human losses (e.g., operation disruption, injuries, fatalities, etc.). As Doebling, Farrar and Prime [1] have mentioned, this increasing interest is not only attributed to economical and time-saving factors, but also to major failures on structures in the past that resulted in the loss of lives. Damages may occur either due to sudden extreme load events (such as strong winds and earthquakes) or due to variable operational loading and environmental effects (e.g., fatigue due to heavy vehicle traffic and corrosion due to environmental conditions). Therefore, it is essential to identify and detect damage at an early stage to maintain the safety and integrity of structures. The use of structural health monitoring (SHM) systems on a regular basis is critical to achieve early damage detection, avoid unpredicted failures, and perform cost-effective maintenance planning.

The vibration-based approach is a subcategory of SHM methods that rely on the fact that structural damage will affect the dynamic characteristics of a structure. The recent trends show an increasing interest in the use of Machine Learning (ML) for SHM systems [2,3,4,5,6,7]. Other methods that rely on Bayesian probabilistic techniques have also been presented in the past [8,9]. The proposed method, however, takes another approach which is based on experimental vibration measurements and FE models. There is no need to acquire large datasets in order to train a Machine Learning model. The only requirement is the development of an optimal FE model, which has a high correlation with the dynamic responses of the physical structure.

The proposed method adopts the model updating technique using optimization algorithms. Similar methodologies have been developed in the past, even before 2000, by various researchers [1,10,11,12,13]. These previous methodologies relied on simplified FE models, most of them using beam elements and a large number of optimization parameters, such as the stiffness of each element to locate the damage in truss-like structures. The simplified models have the advantage of low computational costs. The most recent works followed this approach, such as Chou and Ghabousi [14] using Genetic Algorithms in 2D truss structures. Jafarkhani and Masri [15] have used the CMA–ES evolutionary algorithm coupled with a simple FE Model of a reinforced concrete bridge to detect the location and quantify the damage, while Ding et al. [16] used the Artificial Bee Colony (ABC) algorithm in a 2D truss structure. Zenzen et al. [17] used the Bat optimization algorithm on beam-like and truss structures. Cancelli et al. [18] used the Particle Swarm Optimization (PSO) algorithm in order to locate damage on a pretension concrete girder. The authors of the above-mentioned literature used only simplified FE models with beam elements for truss-like structures. On the other hand, Nicknam and Hosseini [19], Vo-Duy et al. [20], and Baghi [21] have incorporated FE models with plate elements into their procedures to detect damage. Two review articles [22,23] can give a broader view to the reader on the specific subject, mentioning in more detail the algorithms that have been used, different metrics, strategies, and methods.

One common characteristic of all the above-mentioned literature is the working principles of the model-updating procedure in order to locate damage in a structure. Researchers of these past works have employed optimization algorithms with a large number of search parameters, e.g., by updating the stiffness of each element or groups of elements of the complete structure simultaneously. This has lead to a change of the global stiffness matrix. At the end of the procedure, the updated stiffness matrix revealed the difference of each element stiffness and, thus, the damage could be located and/or quantified. The disadvantage of this methodology is seen when considering a large and complex finite element model. It might consist of 2D and 3D elements, where the number of parameters increases, resulting in a dramatic increase in the cycles of each optimization algorithm required for the final result. This leads to a parallel increase of the computational cost.

The approach presented in the following sections has the advantage of a fixed number of search parameters (dimensions of the search space), thus, only the computational cost of the FE model itself affects the performance between different structures. This is achieved by changing only a submatrix of the stiffness and mass matrices to identify the damage. More specifically, a parametric area is inserted into the FE model changing stiffness and mass to simulate the effect of the real damage in a physical structure. This area is controlled by the metaheuristic optimization algorithm, in this case, the Particle Swarm Optimization (PSO) algorithm [24], that is embedded in the proposed damage-detection framework. The framework uses four (4) locational parameters along with two (2) more parameters controlling the material properties of the specific damaged area. The parameters, six (6) in total, can be reduced only if the structure is expanding at a specific axis or plane. A similar approach has been also demonstrated by Gomes et al. [25], where the authors altered the material properties only locally, meaning that only a submatrix of the stiffness matrix was changing during the optimization procedure. Their experimental setup consisted of a single Carbon Fiber Reinforced Polymer (CFRP) composite plate while using Genetic Algorithms (GA) and modal analysis to localize the damage.

Besides the fixed number of the search space dimensions, the proposed method also has the advantage of requiring only output experimental data, while the input excitation does not have to be recorded. This is achieved with the use of the Transmittance Functions (TFs) [26] as a measure of comparison between the physical structure and the FE Model. Compared with other metrics, such as the Power Spectral Density (PSD), the TFs have shown to be prone to structural damage [27] and they can be easily calculated between two different acceleration signals without any information about the input excitation. Other promising metrics also exist in the literature, such as the spectral kurtosis [28], while based on the literature it is mostly used in structures with rotating components.

A valid concern in any model-based damage-detection method is the accuracy of the FE model to simulate the dynamic response of the real structure. A wide variety of existing optimization algorithms can provide a solution to this problem and improve the accuracy of the FE model. For example, the above-mentioned optimization algorithms, which were configured for the damage-detection problem, can be applied successfully in a model-updating scheme to minimize the error between a real-world structure and an FE model. In order to develop the optimal FE model for the use of the proposed method, the Covariance Matrix Adaptation–Evolution Strategy (CMA–ES) algorithm [29] was selected that has been applied successfully at FE updating problems [30,31] in the past with linear and non-linear FE models. Transmittance Functions are also used in this work to obtain the optimal FE model, i.e., the FE model of the healthy structure is updated in an attempt to minimize the error between the obtained numerical Transmittance Functions and the experimental Transmittance Functions.

The proposed damage-detection framework can be applied to complex structures consisting of multiple parts and different materials. The core of the framework consists of a properly configured metaheuristic algorithm while a requirement to apply the framework is an accurate FE model of the structure. When damage occurs on a physical structure some non-linear local behaviors might appear and while the framework is using only linear behavior on the modeled materials it can overcome some small non-linearities within limits. Additionally, this implementation can be applied in detailed FE models that might contain beam, shell, and solid elements. As it was also mentioned [32], these types of damage-detection systems do not need to locate and/or quantify the damage with extreme accuracy. As such, the size of the inserted damaged area on the optimal FE model does not represent the exact size of the damage, but, rather, contains the damage on the physical structure. This size may vary depending on the size or the complexity of the structure but also on the extent of the damage, which can be observed by the experimental measurements.

In conclusion, in comparison with similar past research, the novelty of the proposed method relies upon the following. First, it is a model-based damage-detection method using vibrational measurements with output-only information. Second, it can be applied to detailed FE models of any shape and structure with multiple parts and different materials. And, third, with a properly configured optimization algorithm, only a fixed number of six optimization parameters are needed to locate the damage in any structure with accuracy using a detailed FE model.

The presentation of this work is organized as follows. Section 2 describes the meta-heuristic optimization algorithms which are used in the context of this work and their implementation on the SHM method. Section 3 presents the background of the proposed damage-detection methodology with the use of optimization algorithms coupled with an FE model and experimental measurements. Furthermore, the use of Transmittance Function is presented, which is used as a metric of comparison between the real-world structure and the FE model. The choice of the objective function is also discussed which is a significant step for the successful application of the proposed methodology. Following on, the effectiveness of the proposed methodology is validated in Section 4 via two illustrative examples. First, a simulated small-scale model of a laboratory-tested vehicle-like structure, and, second, a real experimental CFRP composite beam structure. In the first validation example, two damage cases are examined, one with an inserted crack and one with an added mass, while the accuracy of the delivered predictions is explored using simulated, noise-contaminated data, as well as modally reduced models. In the second example, a real experimental CFRP composite beam structure is presented, developing, first, an optimal FE model from experimental measurements of the healthy structure. Similar damage scenarios were examined, one with a small added mass and one with a local reduction of stiffness on the CFRP beam. The robustness of the proposed method is achieved by using random excitations. The final results demonstrate that the proposed damage-detection framework is highly promising, as the damage prediction was successfully performed on both structures with the location of the physical damage always within the predicted damaged area. Finally, the conclusions are summarized in Section 5.

## 2. Metaheuristic Algorithms

In this paper, two metaheuristic algorithms are used for different purposes. The first step is to develop an optimal Finite Element Model that can accurately describe the dynamic response of the examined structure in the frequency range of interest. For this task, the Covariance Matrix Adaptation–Evolution Strategy (CMA–ES) algorithm is used to finely tune the structure’s model in conjunction with experimental measurements from the healthy structure. The core of the damage-detection framework uses the Particle Swarm Optimization (PSO) algorithm with proper variable selection in conjunction with experimental measurements from the damaged structure, as will be presented in the following sections.

The current FE model-updating procedures in the context of this work are employed and tested with linear FE models. While the optimization problem itself has linear continuous search parameter boundaries, other algorithms can be used for both tasks but with proper selection. While, for the first task, it is not trivial to select an optimization algorithm that provides an FE model-updating scheme, for the second task, a more careful selection of algorithm is needed. For example, both CMA–ES and PSO can perform in the presented damage-detection framework in a simple single-part model. But, in a more complex model consisting of multiple parts and multiple materials, the CMA–ES could not perform as intended due to its selection and sampling mechanism.

### 2.1. Covariance Matrix Adaptation–Evolution Strategy (CMA–ES)

The CMA–ES [29,33] is a general-purpose population-based, stochastic, and derivative-free algorithm. In the past, it has been applied successfully to FE updating problems with linear and non-linear FE models [30,31,34] and has demonstrated rapid convergence capabilities, especially when searching for global optimum values. A brief explanation of CMA–ES main steps can be found in Algorithm 1.
**Algorithm 1.** Main steps of CMA–ES algorithm.1. Initialize distribution parameters 2. **While** termination criterion is not met **do** 3. Sample population from the multivariate normal distribution 4. Evaluate the objective function for each parameter set 5. Update the multivariate normal distribution based on a percentage (50% in this case) of the best parameter sets 6. **End** 7. The optimal solution is found for the parameter set that corresponds to the minimum objective function

A free distribution of the CMA–ES algorithm is used in the present work. It is implemented within Π4U framework [35] based on a state-of-the-art task-parallel library for clusters, called TORC [36]. This library is designed to provide unified programming and runtime support for computing platforms that range from single-core systems to hybrid multicore-GPU clusters and heterogeneous grid-based supercomputers.

Let $\underline{\theta }\in R$ be a set of parameters that describe the corresponding Finite Element Model. In this case, this set includes the material properties and damping ratios of the examined structure. Consider $g\left(\underline{\theta }\right)$ being the model prediction, given the values of the parameter set $\underline{\theta }$, while y corresponds to the dynamic experimental measurements. The objective functions for this task, that CMA–ES is called to minimize, can be formulated as the sum of the normalized sum of square errors J.

(1) $$J\left(\underline{\theta }\right)=\frac{1}{n}\sum_{i=1}^{n}\frac{\sum_{j=1}^{m}{\left(g_{ij}\left(\underline{\theta }\right)-y_{ij}\right)}^{2}}{\sum_{j=1}^{m}{\left(y_{ij}\right)}^{2}}$$

As the transmittance functions being used for the damage-detection framework, $g(\underline{\theta }),\;y$ correspond to the FE model’s and experimental Transmittance Functions with the subscript *j* to state the frequency step and *i* the Transmittance curve. The total number of Transmittance Functions is n that includes all the combinations (unique only) between the acceleration sensor measurements in their respected axis, while m is the total number of frequency steps.

At the end of this task, the parameter set $\underline{\theta }$ that corresponds to the minimum of Equation (1) is used to describe the optimal Finite Element Model. It must be noted that to acquire the optimal Finite Element Model other measurements, such as acceleration in the time domain or FRFs, can also be used.

### 2.2. Particle Swarm Optimization (PSO) Algorithm

The Particle Swarm Optimization (PSO) algorithm is employed as the core of the damage-detection framework. It is a population-based algorithm that belongs to the subarea of Swarm Intelligence in the Computational Intelligence category. It was initially introduced by Kennedy and Eberhart [24,37], while it was inspired by the social behavior of animals, such as a flock of birds, and it was initially presented as a method for continuous optimization, such as optimizing the weights of a neural network. Because of its effectiveness and the fact that it was based on a very simple concept while requiring only basic mathematic operations, a large number of variants have been introduced over the years, such as the Inertia Weight PSO [38], Fully Informed PSO [39], Adaptive PSO [40], Adaptive Hierarchical PSO [41], Comprehensive Learning PSO [42], Evolutionary PSO [43], and even hybrid optimization schemes, such as GA–PSO [44] which is a mixture of Genetic Algorithm and the PSO.

For the present work, the simple initial variant of the PSO [24] has been implemented in Matlab, which also takes into consideration the Inertia Weight [38]. The initial swarm of the PSO is consisting of the generated particles/population (parameter set) of the optimization algorithm. On any given iteration the objective function is calculated for each particle. As will be described in more detail later, the behavior of each particle during the optimization is influenced by all or a portion of the other particles of the swarm. In order to control this behavior, two different versions of the algorithm have been proposed [37]. The first is the GBEST version, in which the best solution from all particles is influencing the velocities of all the other particles. The second, which is the one used in this work, is the LBEST version. In the LBEST version, the velocity of each particle is affected by the inertia, its own personal best value, and, instead of the global best value (GBEST), it is affected by the best solution from a number of its nearest particles (neighborhood).

Consider a continuous k-dimensional search space, where k represents the number of variables to be used for the evaluation of the objective function G, $G:\;{\mathbb{R}}^{k}\to \mathbb{R}$. At first, the initial swarm (population), $Pop=\{p_{1},p_{2},\ldots ,p_{n}\}$, which consists of n number of particles, $p_{i}\in {\mathbb{R}}^{k}$ for i=1,2,…,n, is created randomly from a uniform distribution. Each particle $p_{i}$ can be described by its position vector $x_{i}^{t}$, $x_{i}\in {\mathbb{R}}^{k}$, for the given time step t, and its velocity vector $v_{i}^{t}$, $v_{i}\in {\mathbb{R}}^{k}$. As was mentioned, the particle is affected by its own personal best value of the objective functions, so let $pbest_{i}^{t}$, $pbest_{i}^{t}\in {\mathbb{R}}^{k}$, be the vector that remembers the set of parameters that correspond to the minimum value of the objective function that the particle i has found.

The particle’s velocity is also affected by its neighborhood best values (local best values), considering N (N<n) to be the number of neighborhood particles, or, better, to be a fraction of n. In case N=n (or in the case N is a fraction of *n*, N=1) then the GBEST version of PSO is used. Again let $lbest_{i}^{t}$, $lbest_{i}^{t}\in {\mathbb{R}}^{k}$, be the vector that remembers the set of parameters that correspond to the minimum value of the objective function that has been found from all the particles that are contained inside the neighborhood of particle i.

When the evaluation of the objective function is completed for every particle, the velocities and positions are updated according to the following rules:

(2) $$v_{i}^{t+1}=\underset{\mathrm{Inertia}}{\underset{︸}{w\cdot v_{i}^{t}}}+\underset{\mathrm{Cognitive}}{\underset{︸}{c_{1}\cdot R1_{\left(i,\;i\right)}\cdot (pbest_{i}^{t}-x_{i}^{t})}}+\underset{\mathrm{Social}}{\underset{︸}{c_{2}\cdot R2_{\left(i,\;i\right)}\cdot (lbest_{i}^{t}-x_{i}^{t})}}$$

(3) $$x_{i}^{t+1}=x_{i}^{t}+v_{i}^{t+1}$$

where w is the inertia weight and $c_{1},c_{2}$ are the acceleration coefficient which are all defined prior to the start of the algorithm and, in this variation of PSO, do not change during the optimization. R1, R2 are two k×k diagonal matrices with diagonal elements sampled at each iteration from a uniform random distribution with values from 0 to 1.

From Equation (2), it is clear that the Inertia term carries the particle into its previous direction, the Cognitive Part is the force that pulls the particle towards its personal best position and the Social Part is the force that drags the particle towards the best positions known from its neighborhood particles [45].

The inertia weight and acceleration coefficients, $\{w,\;c_{1},\;c_{2}\}\;\in \mathbb{R}$, are chosen prior to the start of the optimization and greatly affect the ability of the algorithm to find the global best solution and the computational cost until the termination criteria are met. The values used for the PSO’s internal parameters are referred in Table 1. While these values have been found to be applicable to the current problem other configurations could also exist.

The objective function, G, that is used for the damage-detection framework is presented with details in Section 3, which follows. Algorithm 2 is summarizing the main steps of the Particle Swarm Optimization algorithm. It must be noted that the algorithm allows for parallel evaluation of the objective function for all particles at the current cycle. This task was implemented in Matlab taking advantage of multi-core and multi-thread capabilities of modern processors while minimizing the execution time until termination. The computer that was used hosts two (2) Intel^®^ Xeon^®^ Gold Processors 6130 (22 M Cache, 3.70 GHz) with 16-cores and 32-threads, resulting in a total number of sixty-four (64) logical (virtual) cores and 128 GB of RAM, on the Linux Ubuntu 18.04 Operating System which is distributed by Canonical, London, UK.
**Algorithm 2.** Main Steps of the PSO Algorithm.
1. Set *w*, *c*_1_, *c*_2_, *n* and variable bounds 2. Randomly generate the initial swarm while enforcing the variable bounds 3. **While** termination criterion is not met **do** 4. **for** each particle *i* **do** 5. Evaluate the objective functions 6. **if**
$G(p_{i}^{t})<G(pbest_{i})$
**then**
$pbest_{i}\leftarrow p_{i}^{t}$ 7.
$lbest_{i}^{t}=\min(pbest_{\mathrm{neighbors}}^{t})$ 8. Update velocity, Equation (2) 9. Update position, Equation (3), while enforcing the variable bounds 10. **End** for 11. **End** while 12. The optimal solution is found as the parameter set that corresponds to the minimum objective function

## 3. Damage-Detection Framework

### 3.1. Description of the Proposed Damage-Detection Framework

Consider a healthy real-world structure that could be described by S and let **M** be the corresponding Optimal FE Model.

In general terms, the relationship between the real structure S and the Optimal FE Model **M** could be described as:

**M** = ***S*** + *e*_1_ (4)

When damage occurs at the real-world structure the Equation (4) can be transformed as:

**M**_*dam*_ = ***S***_*dam*_ + *e*_2_ ⇒ **M** + *d***M** = ***S*** +d***S*** + *e*_2_ (5)

where ***S***_*dam*_ and **M**_*dam*_ represent the damaged real-world structure and the corresponding damaged FE Model that approximates the response of the structure as closely as possible. Additionally, parameters $e_{1},\;e_{2}$ represent the error between the real-world structure and the FE Model. Parameter $e_{1}$ at Equation (4) represents the error between the FE Model and the real-world structure at their healthy states, while parameter $e_{2}$ at the damaged state. When damage occurs, such as a crack, in the physical structure, there is always the possibility of creating a nonlinear behavior locally. For the present work, a damaged linear FE Model was created to approximate the dynamic behavior of the damaged structure and, thus, the two parameters ($e_{1},\;e_{2}$) are not equal.

The proposed damage-detection framework is using optimization algorithms with proper set-up and only output measurements (accelerations) to find the *d***M** that is highly correlated with dS. While dS can be described as the change in the structure, since the healthy experimental measurement, or, in other words, the damage on the structure, *d***M** is the change that the Optimal FE Model, **M**, must make to better describe the $S_{dam}$. Due to the modeling error, *d***M** is never exactly equal to dS but it is the closest approximation possible while using a linear FE Model. In this framework, *d***M** includes the location of the damaged area and an approximation of the change of material properties at this area in terms of Elastic modulus and density. These material properties cannot quantify the damage due to modeling errors, but they can provide some insight to the type of the damage, e.g., loss of stiffness in case of a crack.

In other words, the proposed framework searches for local changes at the material properties of the FE Model while trying to find the parameters that better describe the dynamic experimental measurements. The accuracy of the location depends on the accuracy of the Optimal FE Model compared with the healthy structure.

The equation of motion, Equation (6), can be used to describe the Optimal Finite Element Model (**M**) of the examined structure.

(6) $$M\ddot{x}+C\dot{x}+Kx=F$$

where F is the external excitation and $\ddot{x},\;\dot{x},\;x$ represent the acceleration, velocity, and displacement vectors, M, C, K are the Mass, Damping, and Stiffness matrix, accordingly, and so the model can be fully described by **M**(**M**, **C**, **K**). Assuming that damage in a structure will affect the Mass and Stiffness matrices, the major target is to find the appropriate dM and dK that results in the corresponding *d***M** and so the **M**_*dam*_ that approximates the damaged structure $S_{dam}$.

To achieve this goal a population-based metaheuristic algorithm is used, in this case, the Particle Swarm Optimization, properly configured for this specific task. The PSO handles the task to search for the optimal solution through the entire search domain. This domain includes a total of six parameters with the first two representing the percentage change of the Elastic Modulus and Density, $\left\{p_{E},p_{D}\in \mathbb{R}:(0,UB]\right\}$, where *UB* is the selected upper bound while when $p_{E},p_{D}=1$ the material properties remain the same. The other four parameters control a parametric damaged area that is inserted into the FE Model and moves into the complete three-dimensional space. More specifically, this area is controlled by its central element. Let L(P,X,Y,Z) be the vector describing the central element of the damaged area where *P*,$\left\{P\in \mathbb{R}:[0,1]\right\}$, represents the part of the FE Model in a multi-part structure and X,Y,Z, $\left\{X,Y,Z\in \mathbb{R}:[0,1]\right\}$, corresponding to the local coordinates for the central element expressed as a fraction of the dimension of the specific part chosen by *P*.

After this selection, the framework starting from the central element starts to expand this area in all directions until it reaches a specific number of elements or a specific radius. The size of the area could also be added as a parameter for the optimization algorithm while in the cases examined in the following sections this is not implemented. In cases where the examined structure includes parts with a large difference in size it would be recommended to add the extra parameter.

**Figure 1:**
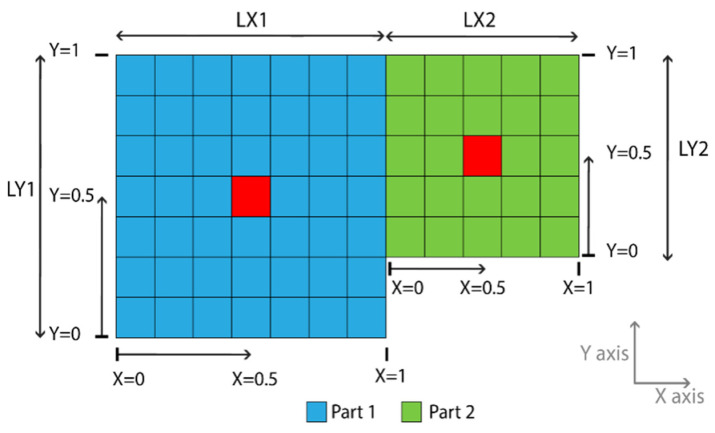
Example of the damaged area center selection in two parts.

For a better explanation of the selection process, consider a two-part model, with different dimensions and materials joined together, as shown in Figure 1.

Both parts have shell elements at the *X*–*Y* axis plane, the *Z* parameter does not affect in this situation and will be set to zero (*Z* = 0). The *P* parameter is defined as:

(7) $$P=\;\;\;\begin{array}{cc} 0\leq P<0.5\;\;\;\to & \mathrm{Part}\;\;\;1\\ 0.5\leq P\leq 1\;\;\;\to & \mathrm{Part}\;\;\;2 \end{array}$$

The two centers (of the damaged area), highlighted in red, are both at the center of each part and can be selected with the following definitions of *L*:

1. For the center at Part 1: L(P,X,Y,Z)→L(0.25,0.5,0.5,0)
2. For the center at Part 2: L(P,X,Y,Z)→L(0.75,0.5,0.5,0)

**Figure 2:**
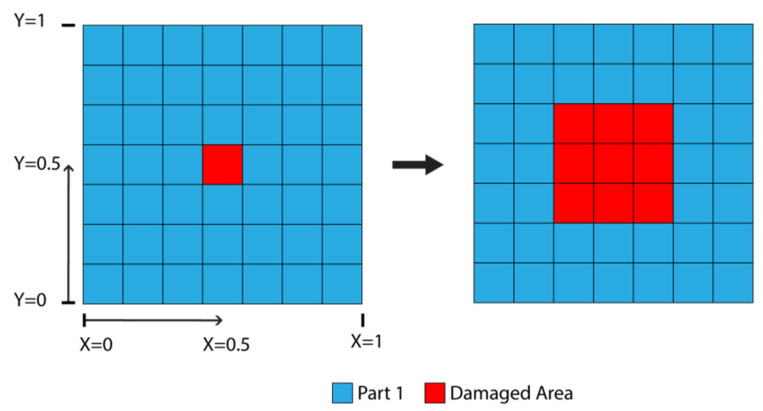
Expansion of the damaged area.

It is obvious that because the X,Y,Z parameters are expressed as a fraction of the selected part in both of the above cases, while they have the same value, the L value points at different coordinates of the global coordinate system. Also, it can be seen in Figure 1 that X,Y,Z parameters are taking the same values regardless of the selected part and the dimensions *LY*1, *LX*1, *LY*2, and *LX*2. If the first case is selected (Part 1), the creation of the damaged area will be completed following the steps below. First, the framework extends the damaged area to any dimension, as shown in Figure 2. For the purpose of this example, the expansion is set to be in one “zone” of neighborhood elements. This makes it easy to create damaged areas on any 3D FE model that uses either 2D shell elements or 3D solid elements. Finally, the material properties of the damaged area are created. The framework was created keeping in mind that a complex structure might consist of parts with different materials.

From the material of the part that is selected each time with the parameter *P*, the new properties are calculated using the $p_{E},p_{D}$ with the following equations.

(8) $${\vec{E}}_{dam}=p_{E}\cdot {\vec{E}}_{part}$$

(9) $$D_{dam}=p_{D}\cdot D_{part}$$

where the subscript *dam* is indicating the material properties (Modulus and Density) of the damaged area and as *part* is the material properties of the selected Part from the parameter *P*. The parameters $p_{E},p_{D}$, as have been mentioned before, are inside the search domain and controlled by the PSO.

It must be noted that $\vec{E}$ is expressed as a vector in case the material is not a standard isotropic and might have moduli at other directions (such as a Carbon-Fiber Reinforced Composite material). As such, every modulus is changed with the same factor, and this allows the framework to be applicable at every structure regardless of how many different materials are in use.

It is noteworthy to mention that, while the locational parameters (P,X,Y,Z) have a fixed bound range from 0 to 1, the material parameters have no specific upper bound. Their value is highly dependent on the size of the inserted damaged area on the FE model and the extent of the physical damage. Furthermore, the error between the FE Model and the physical structure will affect the corresponding values of $p_{E}$ and $p_{D}$. Assuming that the size of the damaged area of the FE Model is appropriate compared to the larger parts of the structure, an initial suggested range of both the material parameters would be no larger than [0.1, 1.5]. In case the corresponding best values after the optimization have reached the bounds, the damage-detection framework needs to restart with larger upper bounds and/or with a larger damaged area for the FE Model. Such examples of restarting the framework for larger parameter bounds are included in Section 4.

The above details are the result of extended laboratory testing and are suggested by the authors to the researcher who might replicate the procedure. The researcher must keep in mind that the parameters, such as the size of the damaged area, material parameter bounds, but also the mesh of the FE Model, must be evaluated according to the examined structure.

The import, manipulation, and export of the FE Model were implemented in Matlab. After the creation of the new FE Model containing the damaged area a commercial Finite Element Analysis software is called to evaluate the model’s response, which, in this case, the MSC Nastran was selected.

### 3.2. Transmittance Function

The Transmittance Function (TF) is expressed as the ratio of the Cross-Spectral (CSD), $S_{rs}$, over the Auto-Spectral Density (PSD), $S_{rr}$, between two vibration response signals calculated from Equation (10).

(10) $$T_{rs}(\omega )=\frac{S_{rs}(\omega )}{S_{rr}(\omega )}=\frac{{\ddot{x}}_{r}(\omega )\;\;{\ddot{x}}_{s}^{*}(\omega )}{{\ddot{x}}_{r}(\omega )\;\;{\ddot{x}}_{r}^{*}(\omega )}$$

As $\ddot{x}(\omega )$ is the Fourier transformation of the acceleration signal, with ω to be the frequency. Furthermore, the ${\ddot{x}}^{*}(\omega )$ is the complex conjugate of $\ddot{x}(\omega )$ and subscripts *r*, *s* denote the degrees of freedom on the structure.

As the calculation of TFs does not require the measurement of the input excitation, a change at the TF curve represents a change of the structural properties and is a sensitive method to be used for damage detection [26,46].

If a q number of acceleration signals is used, then Equation (10) can be written as a matrix of all possible combinations as:

(11) $$T={\left[\begin{array}{cccc} 1 & T_{12} & \cdots & T_{1q}\\ T_{21} & 1 & & T_{2q}\\ \vdots & & \ddots & \vdots \\ T_{q1} & T_{q2} & \cdots & 1 \end{array}\right]}_{q\times q}$$

It is not necessary for the complete matrix from Equation (11) to be calculated and only unique combinations need to be used. In previous works in the literature that used TFs on experimental measurements only, the sequential TFs were used for damage detection [26,47,48] that corresponded to the first upper diagonal of the T matrix from Equation (11). For the context of this damage-detection framework, all the upper triangular part of the T matrix is being used that contains all the unique combinations between the acceleration signals. Moreover, at a structure expanding on all three dimensions in the general case that triaxial accelerometers are used, three TF matrices are calculated that are axis-specific on the global coordinate system of the structure. For example, if w is the number of triaxial acceleration sensors then q=3×w and the TF matrices for the *X*, *Y,* and *Z* axis (global coordinate system) can be expressed as:

(12) $$T^{X}={\left[\begin{array}{cccc} 1 & T_{12}^{X} & \cdots & T_{1w}^{X}\\ T_{21}^{X} & 1 & & T_{2w}^{X}\\ \vdots & & \ddots & \vdots \\ T_{w1}^{X} & T_{w2}^{X} & \cdots & 1 \end{array}\right]}_{w\times w}T^{Y}={\left[\begin{array}{cccc} 1 & T_{12}^{Y} & \cdots & T_{1w}^{Y}\\ T_{21}^{Y} & 1 & & T_{2w}^{Y}\\ \vdots & & \ddots & \vdots \\ T_{w1}^{Y} & T_{w2}^{Y} & \cdots & 1 \end{array}\right]}_{w\times w}T^{Z}={\left[\begin{array}{cccc} 1 & T_{12}^{Z} & \cdots & T_{1w}^{Z}\\ T_{21}^{Z} & 1 & & T_{2w}^{Z}\\ \vdots & & \ddots & \vdots \\ T_{w1}^{Z} & T_{w2}^{Z} & \cdots & 1 \end{array}\right]}_{w\times w}$$

Of course, other configurations can exist if, for example, a mix of single-axis and triaxial acceleration sensors are used, so, in this case, the $T^{X},T^{Y},T^{Z}$ matrices are not equal in dimensions.

The use of TFs allows the application of this framework to be applied without the measurement of the input excitation and using only the output information. For the corresponding FE Model, an excitation is needed, and the experimental excitation can be used if it is available, but an artificial random excitation may also be used as it will not affect its TFs.

### 3.3. Objective Function

A black box optimization scenario is employed where the goal is to minimize the Objective Function, $G:\;{\mathbb{R}}^{k}\to \mathbb{R}$ where k is the search space dimension, and the only accessible information is the Function’s values of evaluated search points [29]. Thus, choosing a proper objective function is crucial to effectively apply the damage-detection framework.

The Pearson correlation coefficient is used to compare the experimental Transmittance Functions and the corresponding FE Model Transmittance Functions. It is a measure of the linear correlation between two sets of data and, as a normalized measure, it takes values between −1 and 1. The Pearson Correlation Coefficient can be calculated from Equation (13) for two equal-length sets of data, *A* and *B*, where N is the length of the data sets and μ, σ are the mean value and standard deviation of each set, *A* and *B*, accordingly.

(13) $$Pearson\;Correlation\;Coefficient:\;\;\rho (A,B)=\frac{1}{N-1}\sum_{i=1}^{N}\left(\frac{A_{i}-\mu_{A}}{\sigma_{A}}\right)\left(\frac{B_{i}-\mu_{B}}{\sigma_{B}}\right)$$

A value of ρ=1 indicates that there is a perfect linear correlation between the two data sets, while, on the other hand, ρ=−1 indicates a negative linear relationship and ρ=0 indicates that there is a nonlinear relationship between the two sets but without providing any further details on their relationship.

In this minimization problem, the best available linear relationship is attempted to be found between the Experimental Transmittance Functions and the FE Model Transmittance Functions. So, the error of the Pearson Correlation Coefficient is used, or, as it has also been mentioned as, the Pearson Distance [49], and it becomes obvious from the Equation (14) that since the ρ has a range of [−1, 1] the Pearson Distance has a range of [0, 2], with zero to indicate the perfect linear correlation.

(14) $$Pearson\;Distance=1-\rho \left(A,B\right)$$

Expressing now the Objective Functions, G, in the general form, at Equation (15), as the mean value of all the Pearson Distances between the experimental measurements from the damaged structure and the Optimal FE model. Where $TF^{DEXP}$, $TF^{FE}$ are the Experimental Transmittance Functions (damaged structure) and the FE Model’s Transmittance Functions, while *A* is the total number of Transmittance Functions used with *i* = 1, 2, …, *A*.

(15) $$Objective\;\;Function,\;\;\;\;\;\;\;\;G=\frac{1}{A}\sum_{i=1}^{A}\left[1-\rho \left(T_{i}^{DEXP},T_{i}^{FE}\right)\right]$$

Damage, such as a crack in a part of a real structure, adds a discontinuity and it might not be able to always be modeled with precision as a reduction in stiffness in a continuous area in an FE model. The use of the Pearson Coefficient is forcing an FE Model’s Transmittance Function to take the shape of the damaged Experimental Transmittance Function, but it under-estimates the magnitude difference that could be created due to this discontinuity at the peaks of the curve. This is also the reason why the same objective function is not used to obtain the optimal FE model and Equation (1) was used instead, as the damping ratios may not be evaluated correctly by Equation (15).

The general idea in such optimization procedures is that the more information is available the better the outcome. But in this case, the inclusion of “bad” information could sometimes lead to a wrong estimation of the damage location. The limited-value range of the Pearson coefficient can assist in an initial automated filtering of the information (response signals, in this case). Before the framework is applied, the Optimal FE Model must be evaluated and compared with the healthy structure. From the experimental measurements of the healthy structure, one must compute the Transmittance Functions and evaluate the Pearson coefficient with that of the FE Model. This way, the quality of the optimal FE Model can be evaluated. From the complete set of Transmittance Functions, a subset with the best correlation can be used while the remaining TFs will be disregarded.

**Figure 3:**
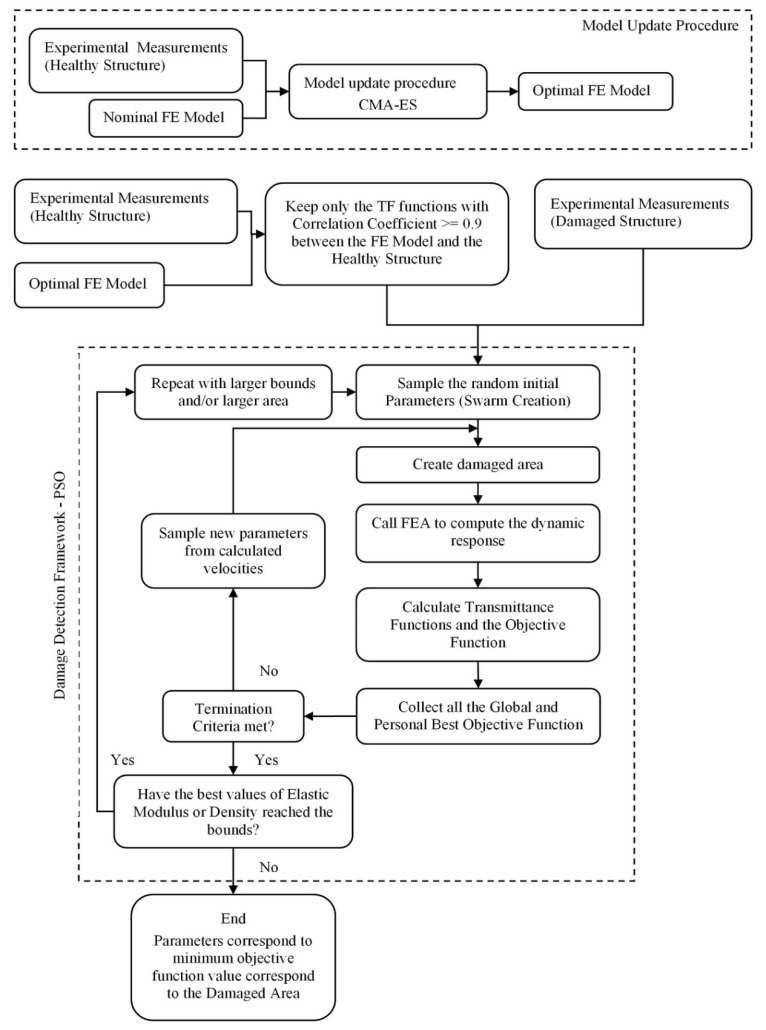
Flow-chart of the proposed damage-detection framework.

The flow-chart of the entire proposed damage-detection framework along with the model-update procedure is presented in Figure 3.

For the present work, this limit is set to ρ≥0.9 (or else a Pearson  Distance≤0.1), meaning the subset of Transmittance Functions that will be used for damage-detection framework will contain only the Functions of the Optimal FE Model with high correlation (ρ≥0.9) with the initial Transmittance Functions of the healthy structure while all the others will be rejected. This limit was set after evaluating different cases in a laboratory environment and can change depending on the required accuracy of the framework or the complexity of the structure.

## 4. Results and Discussion

To illustrate the effectiveness of the proposed technique for damage detection, two applications are considered. The first employs a small-scale laboratory-tested vehicle body-suspension system using simulated damage scenarios that present the general case of application in complex structures consisting of multiple parts. The second one consists of an experimental cantilever CFRP composite beam with glued aluminum connectors.

### 4.1. Application on Complex Structures. Simulated Small-Scale Laboratory-Tested Vehicle Body-Suspension System

**Figure 4:**
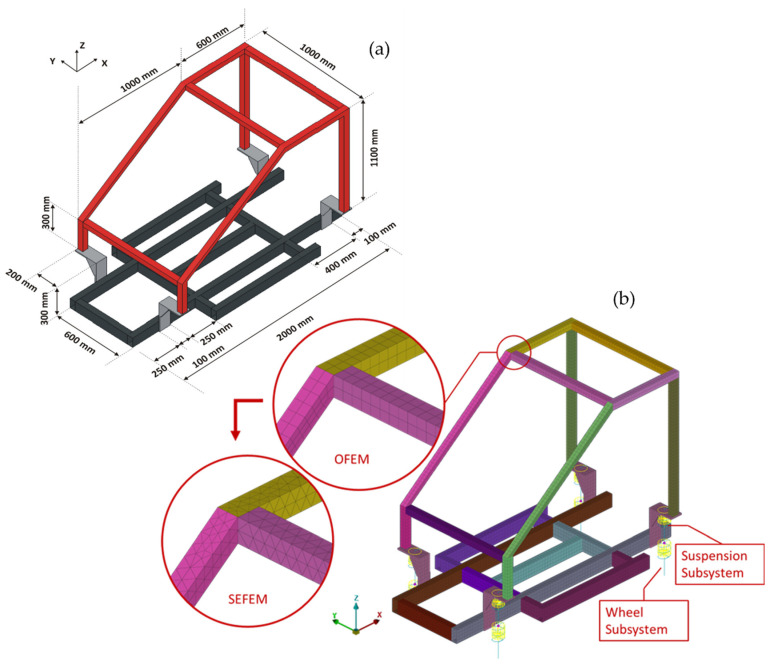
(**a**) Dimensions of the frame substructure Optimal and (**b**) FE Model (OFEM) and Simulated Experimental FE Model (SEFEM).

A small-scale vehicle model, shown in Figure 4, is used to demonstrate, first, the effectiveness of the proposed methodology. This is a laboratory structure, designed to simulate the frame structure of a vehicle on a small scale. Figure 4 presents the geometrical dimensions of the frame subsystem alone. More details of the frame can be found in previous published papers, see [50,51].

In brief, the selected system comprises a frame structure with predominantly linear response and high modal density, consisting of multiple parts, plus four (4) supporting systems with linear or nonlinear actions. These supporting systems consist of a lower set of linear discrete spring-damper units, connected to a concentrated mass, simulating the wheel subsystems, as well as of an upper set of linear or nonlinear discrete spring-damper (bushings) units connected to the frame and simulating the action of the vehicle suspension. The current application is employed as a simulation–simulation scenario using artificial damage. For consistency, as shown in Figure 4, the initial unchanged FE model will be referred to as the Optimal FE Model (OFEM). To simulate a percentage of modeling error from the Optimal FE Model, a second FE model was developed by remeshing and introducing material uncertainties to OFEM. This model will be referred to as the Simulated Experimental FE Model (SEFEM). As a result, the difference of the OFEM and the SEFEM in their healthy state is detected in the discretization variability of the same FE model, developed with different elements. The aim is to introduce and simulate additional modelling error between the optimal FE model and a real-world experimental structure.

The Optimal FE Model (OFEM) consists of 7636 shell elements (7632 quadrilateral and four triangular) for the main structure of the vehicle chassis and 592 hexahedral solid elements for the four parts connecting the structure with the suspension subsystems. In the current application the sets of wheel-suspension subsystems consist of linear spring-damper units. It must be noted that using the Transmittance Functions this framework could be applied even at non-linear wheel and suspension subsystems in a real-world structure. The acceleration at the connection points between the suspension and the structure could be measured and then applied as excitation at the optimal FE model without the existence of the wheel and suspension subsystems [50].

The second FE model, which will be referred to as the Simulated Experimental FE Model (SEFEM), was developed to simulate the healthy experimental measurements and the damaged structure. In order to add modeling error between the SEFEM and the OFEM, different mesh was used along with changed materials properties. The SEFEM consists of 11,128 shell elements (4512 quadrilateral and 6616 triangular) while the solid hexahedral elements remained the same, at 592. The stiffness of all the springs of the wheel and suspension subsystems were changed at the range of 3.5 to 10%, the Elastic Modulus was changed at 6.6%, and the density was changed at 1.3%. The complete OFEM compared to SEFEM in an enlarged area is shown in Figure 4.

**Figure 5:**
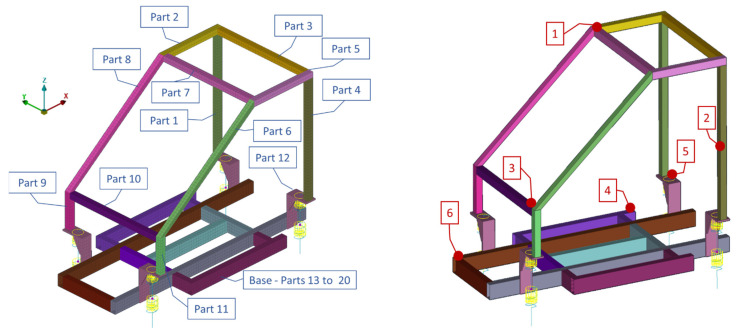
Parts of the FE model (**right**) and acceleration measurement locations (**left**).

The structure consists of 20 parts, as presented in Figure 5, having steel material properties (E=210 GPa, ν=0.3, $\rho =7850\;{\mathrm{Kg}/m}^{3}$). Parts 1–11 have a shell thickness of 2 mm, and parts 13–20 have a thickness of 3 mm. Figure 5 also shows the six (6) measuring locations.

At the current application, all three axes of acceleration measurement are taken into account, thus the complete Transmittance Function table is calculated, based on Equation (12), keeping active all the parameters of the damage-detection framework.

Four (4) different transient displacement base excitations were applied at the wheel subsystems in the vertical direction (*Z*-axis). The displacement excitations were artificially created as a random signal that covered the range of all required frequencies with almost a uniformly distributed power spectrum. The model was solved in modal transient response analysis using the commercial software MSC Nastran. Furthermore, experimental measurements usually contain a level of noise that may originate from different sources, such as the testing hardware or environmental factors, thus, at the acceleration output signals of the SEFEM a 10% noise was added.

From the six (6) locations of measurements, using all three (3) axes of the global coordinate system, eighteen (18) acceleration time histories occur. As so, each of the three (3) tables of Equation (12) have a dimension of 6-by-6 while from the thirty-six (36) Transmittance Functions, included in each matrix, fifteen (15) are used that correspond to the upper triangular part of the matrix containing only the unique combinations between the measurement locations. Furthermore, the total number of TFs from all three axes is forty-five (45), although, as discussed in Section 3.3, a limit must be applied between the OFEM and SEFEM for the sake of location accuracy.

**Figure 6:**
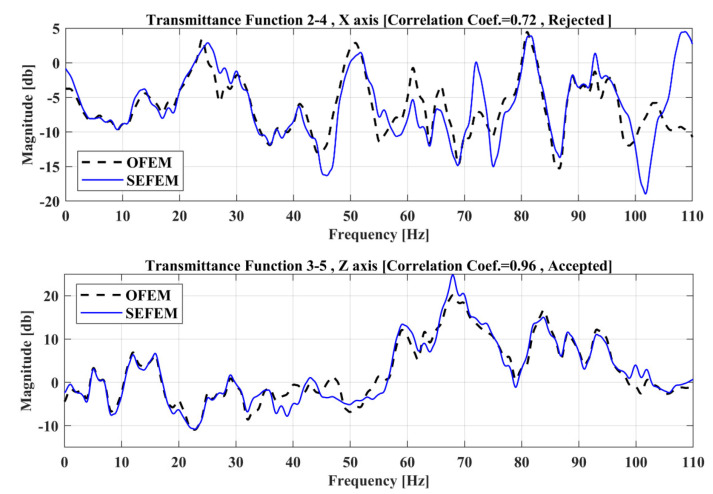
Indicative TFs of the vehicle between the OFEM and SEFEM.

A Transmittance Function curve from the Optimal FE model (OFEM) must have a correlation coefficient, based on Equation (13), higher or equal to 0.9 when compared with the corresponding experimental Transmittance Function (SEFEM) at a healthy state. The calculation of this correlation coefficient is being made prior to any damage on the experimental structure, SEFEM on the present section. This step is ensuring that only TFs with high correlation between the FE model and the experimental structure will be taken into consideration for damage localization. Applying such a threshold, the effective TFs for the damage-detection framework, are limited to thirty (30), while the remaining fifteen (15) do not meet the criterion and are rejected. Two indicative results of rejected and accepted TFs are presented in Figure 6, while the structure is examined at the frequency range of 0–110 Hz.

**Figure 7:**
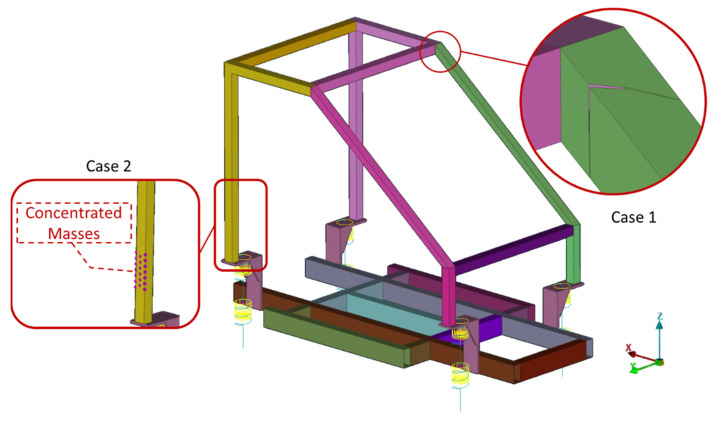
SEFEM damage Cases 1 and 2.

Two damaged cases are examined. At the first (Case 1), a crack is opened at the SEFEM at Part 6 of the structure, simulated with a split between specific elements. In the second case (Case 2), a mass of 1 kg is added at Part 1 of the chassis that corresponds to 0.57% of the total mass of the structure (174.7 kg). The two simulated damage cases of the SEFEM are presented in Figure 7.

A cumulative comparison of the natural frequencies between the SEFEM and OFEM is presented in Table 2. The effect of the introduced model error can be clearly observed in terms of modal natural frequencies in healthy state, along with the respective frequencies of the FE models used to simulate the damaged scenarios. The error from the damaged scenarios is calculated as a per cent error from the SEFEM to express the effect of the damage.

#### 4.1.1. Case 1: SEFEM Crack Damage

**Figure 8:**
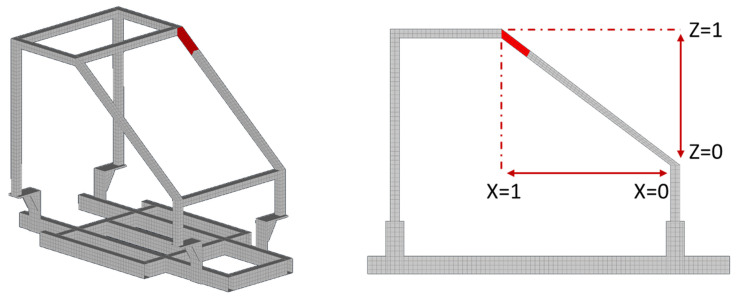
Damaged FE Model of Case 1.

Regarding the first case, the damage is inserted as splitting between the elements at Part 6 near the connection point with Part 7 and Part 5, as can be observed in Figure 7. For the damage-detection framework, the material parameters were set within the range of 0.1 and 1.5. The resulted damaged FE model can be observed in Figure 8 while, in comparison with Figure 7, it is obvious that the damaged area (represented in red color) contains the simulated crack opening of the damaged SEFEM. Furthermore, in Figure 8 a representation of the locational parameters X, Z can be observed when Part 6 is selected.

**Figure 9:**
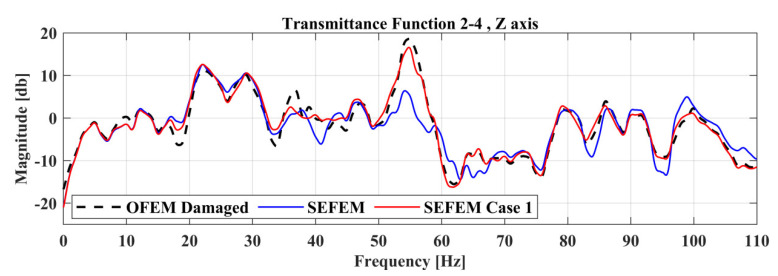
Comparison of TFs between the OFEM, SEFEM, and damaged SEFEM of Case 1.

**Figure 10:**
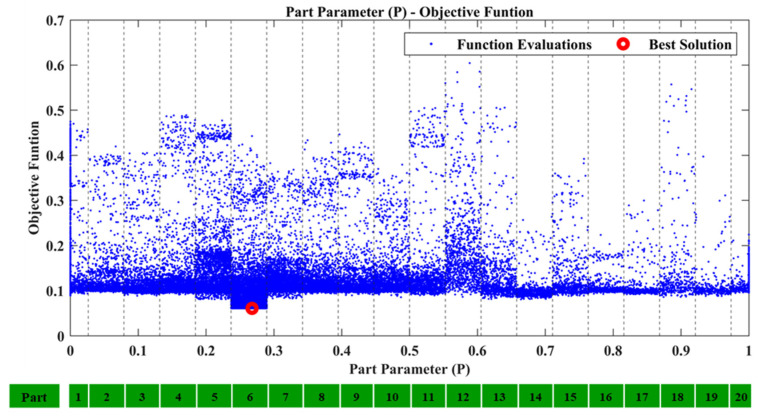
Case 1: Part parameter (*P*) versus the Objective Function values.

**Figure 11:**
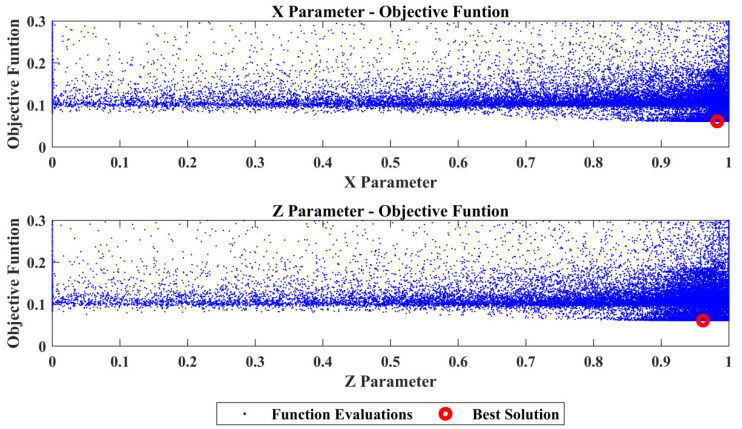
Case 1: *X* and *Z* parameters versus the Objective Function values.

From the comparison of the TFs between SEFEM and the damaged SEFEM one can observe that the damage affects a specific frequency range, which is something that is expected as a similar pattern was observed in Table 2 at the comparison of the natural frequencies. An indicative comparison from the 30 total TFs is presented in Figure 9 which includes the TF, between locations 2 and 4 at the *Z*-axis, of the SEFEM, the damaged SEFEM and the resulted damaged OFEM from the damage-detection framework. The material parameters $p_{E},p_{D}$ that correspond to the best solution were found to have a value of 0.35 and 0.7, respectively. The part parameter (*P*), presented in Figure 10, resulted in a value within the range of Part 6. The specific part extents only at the *X*–*Z* plane (global coordinate system) while along the *Y*-axis its dimensions are negligible, as only the X,Z parameters have an effect on the location of the damaged area within this part. Their values with the resulted objective function value are presented in Figure 11.

#### 4.1.2. Case 2: SEFEM Mass Damage

**Figure 12:**
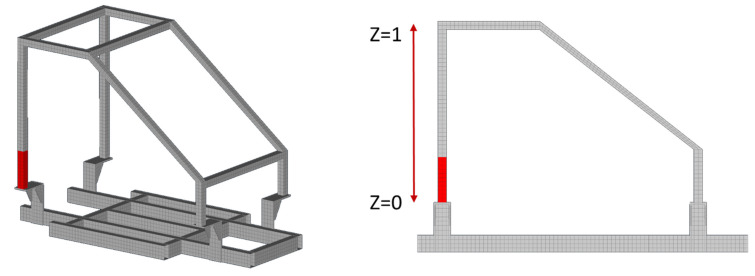
Damaged FE model of Case 2.

The second damage case includes a mass added at Part 1 as a group of 20 concentrated mass elements (CONM2). The total added mass is equal to 1 kg which corresponds to 0.57% of the total mass of the structure which is 174.7 kg. The mass is added at the bottom of Part 1 and, more specifically, at one face of the square hollow section beam. At first, the material parameters at the damage-detection framework were set within the range of 0.1 and 1.5. Upon completion of the first execution, the material parameters have reached the bounds making the result unreliable. During restart, the range was extended with an upper bound of 5 without changing any other parameter. The task was completed successfully with the best solution of the framework to create a damaged area that includes the area of the added mass on the SEFEM. In Figure 12 the resulted damaged FE model of Case 2 can be observed along with the locational parameter Z when Part 1 is selected.

**Figure 13:**
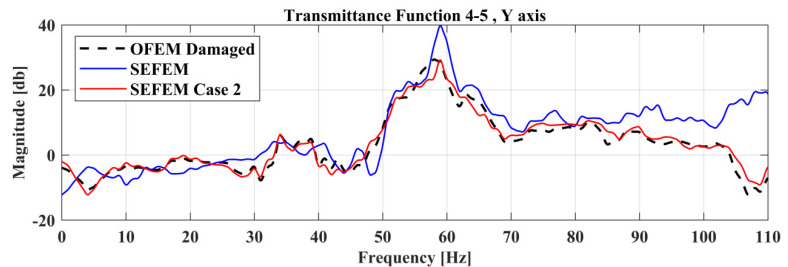
Comparison of TFs between the SEFEM, damaged OFEM, and damaged SEFEM of Case 2.

The effect of the damage at the TF curve can be observed in Figure 13, where TF between locations 4 and 5 at the *Y*-axis, of the SEFEM, the damaged SEFEM of Case 2 and the resulted damaged OFEM from the damage-detection framework is presented. The material parameters $p_{E},p_{D}$ that correspond to the best solution were found to have a value of 3.02 and 3, respectively.

**Figure 14:**
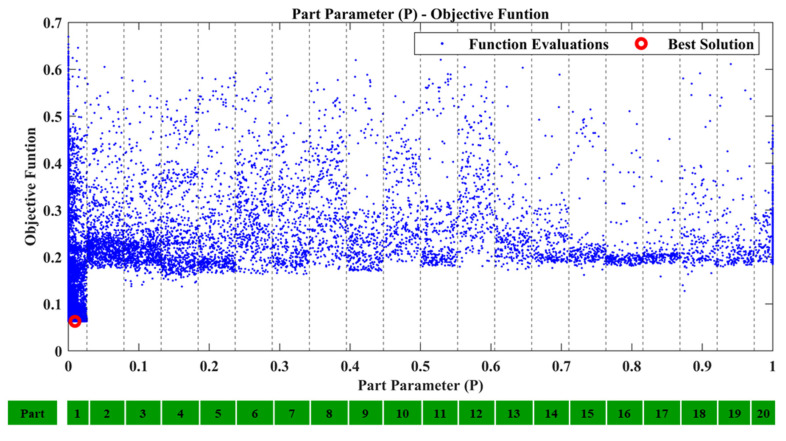
Case 2: Part parameter (*P*) versus the Objective Function values.

**Figure 15:**
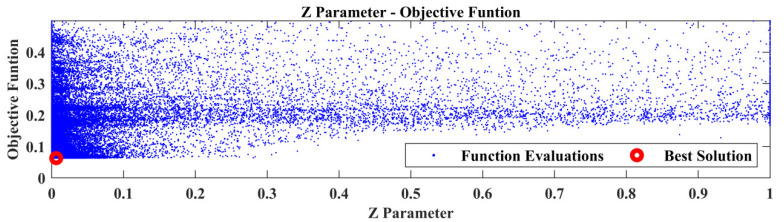
Case 2: *Z* parameter versus the Objective Function values.

The part selection parameter (*P*), as can be observed in Figure 14, corresponds to Part 1 of the structure, while the Z parameter has a value of 0.006. Figure 15 presents the corresponding best solution of the damage-detection framework. For testing purposes only, this task was executed also with the upper bounds of the material parameters equal to 8, but the result remained the same as in the case where the upper bounds were set at 5. While the location of the damaged area is correct, it must be noted that the material parameters do not quantify the damage and the corresponding best values might be different to what was expected. It is noteworthy to say that, in this case, the Elastic modulus parameter is higher than anticipated. This is attributed to model error between the FE model and the “physical” structure, OFEM and SEFEM in this case. Furthermore, the manner of modeling physical damage as a linear continuous area with different mechanical properties imports additional error into the whole process.

### 4.2. Experimental Application on a Cantilever CFRP Composite Beam

The use of composite materials has been growing in the last years in several industries, such as aerospace, aviation, and automotive. Their brittle fracture when subjected to impacts and their damage behavior in general is a complex research topic. There are cases where damage in the material might not be easily located with a visual inspection, such as delamination. Furthermore, in complex multi-part structures, it might be even more difficult to locate the damage.

This type of material is non-homogenous, and its behavior is non-isotropic. The following experimental set-up was chosen as a benchmark for the proposed method in order to also test its abilities in this type of material.

The experimental set-up consists of a circular hollow section CFRP composite beam with 1 m length and wall thickness of almost 1.72 mm including layers with winding angles of 8° and 86°, as presented in Table 3. It was produced with the filament winding method while at the beam’s ends custom-made aluminum connectors were glued using two-component epoxy glue. One connector was fixed and the other was mounted with an electrodynamic shaker using a rod with a diameter of 2 mm.

**Figure 16:**
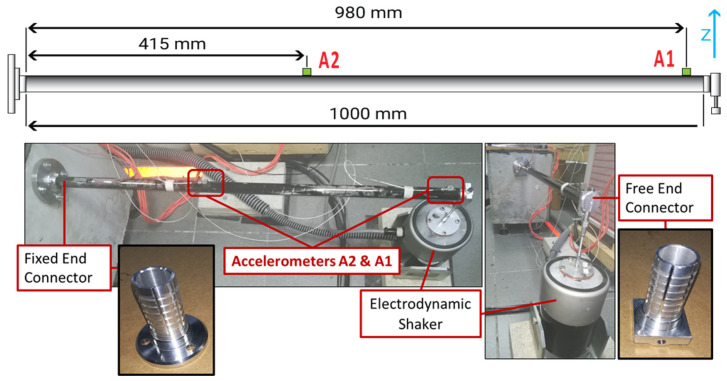
Complete experimental set-up of the CFRP composite beam.

Two triaxial accelerometers were used. Accelerometer 1 was placed at a distance of 415 mm and accelerometer 2 at a distance of 980 mm, both measured from the fixed end of the composite beam. The complete experimental set-up is presented in Figure 16. The first step was to execute experimental measurements from the healthy structure to develop an optimal FE Model. Random excitation was imposed by the electrodynamic shaker along the *Z*-axis at a sampling rate of 5120 Hz. During the experimental process, the signal of the imposed excitation was actually recorded and could be used as raw data for the excitation of the FE Model. Nevertheless, measuring time histories of the imposed excitation is unnecessary because Transmittance Functions needed to implement the presented framework are computed using output-only response measurements.

**Figure 17:**
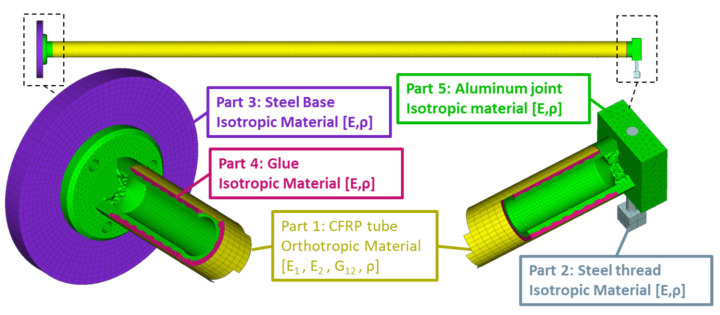
FE Model of the cantilever CFRP beam.

The corresponding FE model is shown in Figure 17. In total 81,433 solid elements were used for the aluminum joints, the steel base, the glue, and the steel thread that connects the free end with the electrodynamic shaker, furthermore, 10,944 shell elements were used for the CFRP composite materials as it is a thin-walled tube. A modal frequency response analysis was selected.

CMA–ES, as described in Section 2.1, was implemented to acquire the twelve (12) parameters, accounting for the material properties of the Optimal FE Model. Transmittance Functions were then used as a measure of fit between the experimental measurements and the FE model predictions. Equation (1) is adopted during this FE model update, at the frequency range from 0–650 Hz. The updated material properties are shown in Table 4, along with the bounds used for the optimization.

**Figure 18:**
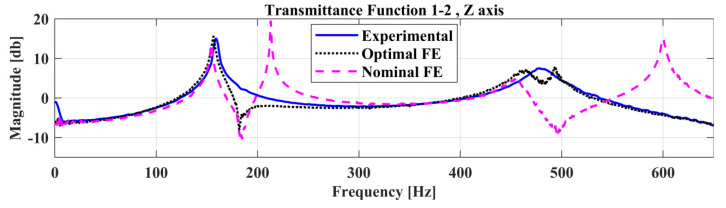
Comparison of the Transmittance Function 1-2 between the experimental measurements, the nominal FE Model and the Optimal FE Model.

A comparison of the experimentally identified natural frequencies with the Nominal and Optimal FE model is presented in Table 5. Furthermore, a comparison of the Transmittance Functions, TF 1-2 at the *Z*-axis, between the experimental measurements, Nominal FE model, and the Optimal FE Model is presented in Figure 18. The Pearson Correlation Coefficient between the optimal FE model and the experimental measurements can be calculated by Equation (13) and is equal to 0.92 which passes the criterion (ρ≥0.9) mentioned in Section 3.3.

**Figure 19:**
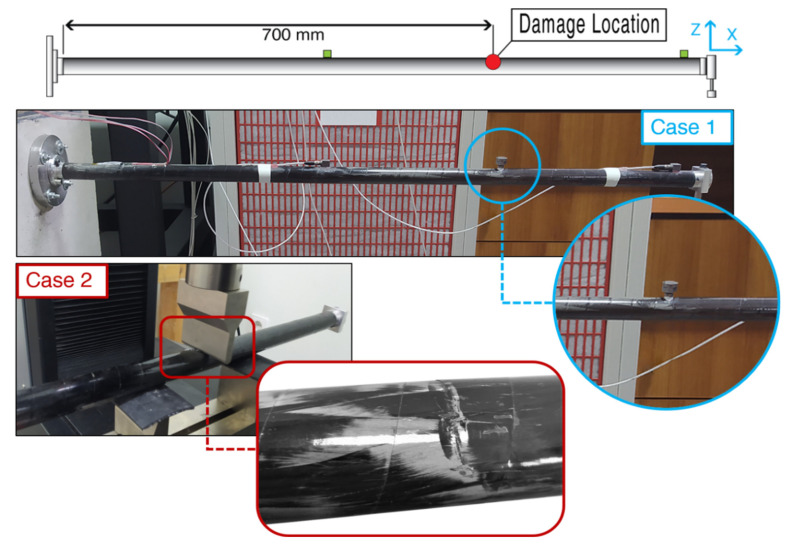
Cantilever CFRP composite beam damage cases.

On the current experimental set-up two damage Cases are examined as presented in Figure 19. Case 1 includes an added mass of 22 g at a length of 700 mm from the fixed end of the composite beam, with the damage corresponding almost 2% of the total structure’s mass (1098 g). Case 2 involves the local reduction of stiffness also at a length of 700 mm from the fixed end of the beam. A compression machine was used in a three-point bending scenario, which presses the damaged location with a total of 1.5 kN forcing the matrix of the composite material to break and create multiple small cracks at the specific location, as shown in Figure 19.

Furthermore, rubber material was placed under the two points that hold the beam, preventing unwanted damage at these points. Two presses were executed, one on the upper side (same side with the accelerometers) and one on the opposite side (180° rotated), both with the same force. After the damage was created small visible cracks could be distinguished, while a slight, local only, deformation was present. In both cases, random excitation was imposed by the electrodynamic shaker during the experimental procedure and an artificial random excitation was generated as input for the Optimal FE Model.

#### 4.2.1. Case 1: Added Mass Damage on CFRP Composite Cantilever Beam

On this specific structure, some parameters of the damage-detection framework described in Section 3.1 are not needed to be included. Only one part is selected to be inspected (the CFRP composite beam), so the P parameter is not used; moreover, as the beam extends mainly at the *X*-axis compared to the other axes it eliminates the need to use all the locational parameters. As a result, three parameters are used in total, i.e., the material parameters $p_{E},p_{D}$, and the X location parameter, while the damaged area introduced into the model is a ring zone with 120 mm in length. The upper bound of the material parameters was set to 2. However, the parameters reached the upper bounds and the framework had to restart. The extended upper bound was set to 8 while the lower bound was retained to 0.1.

**Figure 20:**
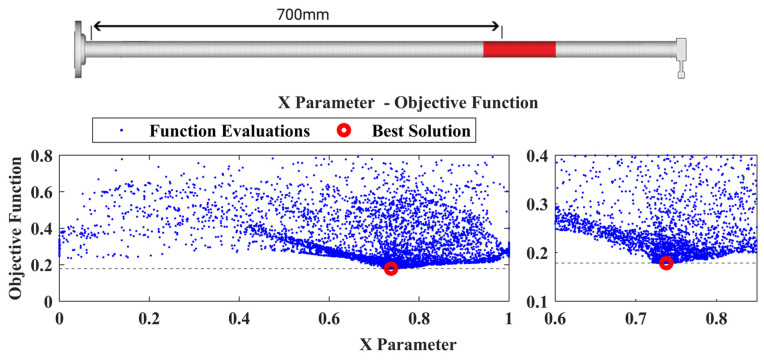
Damaged FE Model along with the location parameter X versus the Objective Function value of Case 1.

**Figure 21:**
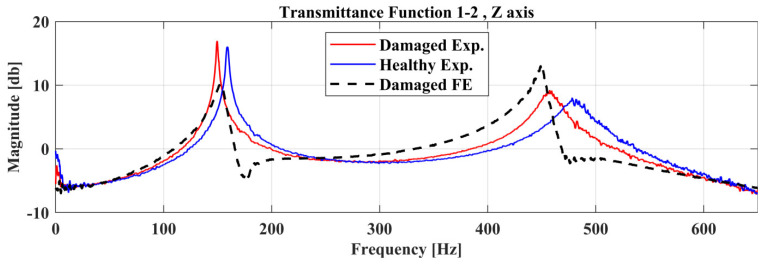
Comparison of the Transmittance Functions 1-2 between the damaged and healthy experimental measurements along with the damaged FE Model.

The damage-detection framework found the best solution that corresponds at an area with its center at X=0.73, as can be observed in Figure 20, while the best material parameters $p_{E},p_{D}$ have a value of 3.18 and 4.7, respectively. The point of added mass is included in this area as presented in the final damaged FE model in Figure 20. Finally, in Figure 21 a comparison of the TFs is presented between the experimental healthy, experimental damaged measurements and the final damaged FE Model.

It is obvious that the added mass on the structure resulted in a shift of the peaks at lower frequencies with the first curve’s peak of the healthy structure to be at a frequency of 159 Hz and shifted to 149.6 Hz for the damaged structure (5.9% change) and the second peak at a frequency of 478 Hz at the healthy structure to be shifted at 457 Hz at the damaged structure (4.3% change). The final damaged FE model has the first peak at 151 Hz, which is a small difference from the 149.6 Hz of the damaged structure (0.93% difference) and the second peak at 454 Hz that is also a small difference from the damaged structure at 457 Hz (0.65% difference). It must be noted that it is not always possible for the magnitude of the damaged experimental Transmittance Functions to be equal to the damaged FE Model using this procedure, as was also explained in Section 3.3.

#### 4.2.2. Case 2: Local Stiffness Reduction Damage on CFRP Composite Cantilever Beam

For the execution of the damage-detection framework, the lower bound of the material parameters was set to 0.1. For the upper bound, considering the initial run and the small difference between the healthy and damaged states, it was set to 1.5. Finally, the length of the damaged area remained the same, as in the previous Section 4.2.1.

**Figure 22:**
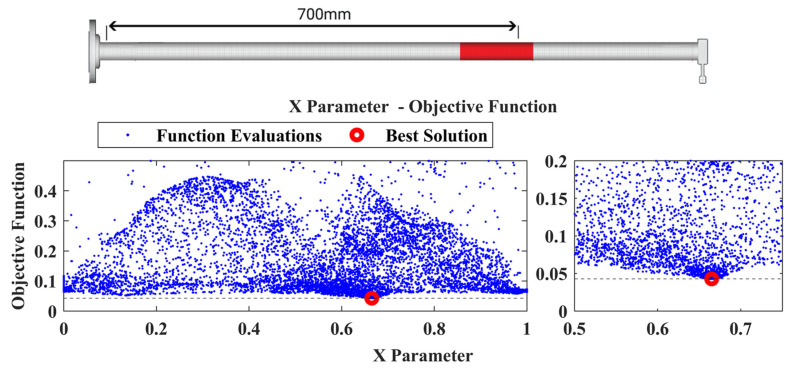
Damaged FE Model along with the location parameter X versus the Objective Function value of Case 2.

The damage-detection framework has found the best solution that corresponds at a location parameter X=0.665, Figure 22, while the material parameters $p_{E},p_{D}$ have a value of 0.3 and 0.7, respectively. The location of the damage lies within the corresponding area of the damaged FE model, as seen in Figure 22.

**Figure 23:**
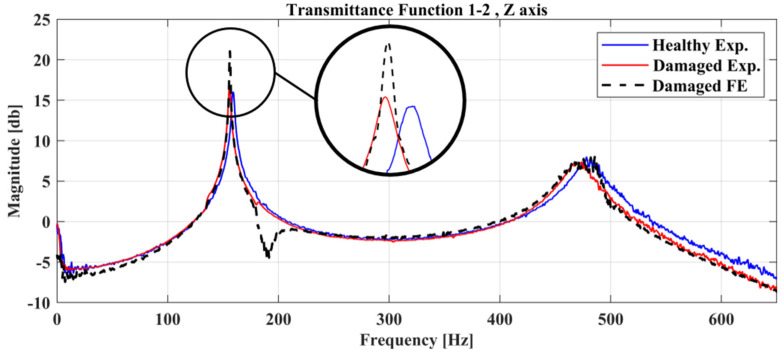
Comparison of the Transmittance Functions 1-2 between the damaged and healthy experimental measurements along with the damaged FE Model.

The comparison between the experimental healthy, experimental damaged measurements and the final damaged FE Model of Case 2 is presented in Figure 23.

The first peak of the experimental healthy state is at a frequency of 159 Hz while the experimental damaged state has been shifted at 155 Hz (2.5% difference) while the second peak is at a frequency of 478 Hz at the experimental healthy state which has been shifted at the frequency of 469 Hz (1.88% difference) at the experimental damaged state. On the other hand, the damaged FE model has its peaks almost in identical frequencies with the experimental damaged measurements. In comparison with Case 1 at Section 4.2.1, the damage of Case 2 has a smaller effect on the dynamic response for the structure.

## 5. Conclusions and Observations

A vibration-based damage-detection framework was presented using optimal finite element modeling, metaheuristic algorithms, and experimental measurements. The primary goal lies in facilitating an optimized vibration-based inspection strategy for the structures by exploiting the value of monitoring information. As a starting point, an optimal FE model is developed that can describe the dynamic response of the healthy structure with accuracy. The optimal FE model along with experimental vibration measurements of the structure compose the inputs of the damage-detection framework, as an output of the framework is a damaged FE Model which approximates the dynamic response of the damaged real-world structure. For this purpose, a parametric damaged area is inserted at the optimal FE model changing stiffness and mass to approximate the effect of the physical damage. Upon convergence of the optimization procedure, the inserted area is highlighting the damaged area of the structure.

The effectiveness of the proposed method and its potential for damage detection is demonstrated via two illustrative examples, a simulated small-scale model of vehicle-like structure and a real experimental CFRP composite beam structure. The robustness of the proposed method is examined using two small damage scenarios for each validation model and combined with random excitations.

The two examined structures introduce different types of difficulties. The first, vehicle-like structure, exposes the framework to a complex multi-part structure. While the second, CFRP composite beam, includes a material with complex damage mechanism and behavior. Each examined case, two per structure, affected the dynamic response of the corresponding structure in different forms and magnitudes. More specifically, the first examined structure, the vehicle-like structure, has a maximum modeling error of 2.38% at the natural frequencies compared with the healthy structure (simulated). The first damage case caused a maximum shift in the natural frequencies of the healthy structure equal to 5.93% and 3.35%. Regarding the second structure, the CFRP beam, the FE model had a maximum modeling error of 2.14% compared with the experimental structure. The first and second damage cases resulted in a maximum frequency shift of 5.9% and 2.5%, respectively.

In conclusion, both examined cases included a maximum modeling error of 2.38 and 2.14% and the damage cases caused changes in the dynamic response of the physical structures from 2.5 to 5.39%. The proposed damage-detection framework was able to complete all four (4) cases successfully and find the affected area, as in all cases the damage of the structure falls within the damaged area of the FE model.

The limitations of this method should also be addressed. The proposed method relies on the accuracy of the FE model, as such the development procedure of the optimal FE model should be a priority as it can determine the accuracy of the predicted damage location. Furthermore, the framework is able to efficiently identify the damage location, but it is not able to quantify it. The major reason is the initial discrepancy between the FE Model and the physical structure. Future research could target the application on systems that exhibit non-linear behavior, as well as to extend through a hierarchical structure to problems of multiple faults.

## Figures and Tables

**Table 1:** PSO Parameters.

Inertia weight, *w* = 0.97	Acceleration Coefficients, *c*_1_ = *c*_2_ = 1.49
Neighborhood, *N* = 0.25 (Fraction of *n*)	Stall Iterations = 20
Termination Tolerance = 1 × 10^−6^	Population, n=100×k *

* where *k* is the number of variables. While this general rule is followed, regarding the application of the damage-detection framework it must be noted that in very complex (with multiple parts and multiple materials) structures and small amounts of damage that have minimal effects on dynamic responses it is advised that a larger population be used. This can be tested with a benchmark test on the specific structure simulating the damage and using FE Model (healthy) to FE Model (damaged) comparison.

**Table 2:** Comparison of the vehicle natural frequencies (Hz).

Modes	OFEM Frequencies	SEFEM Frequencies	Error (%)	SEFEM Case 1	Error from SEFEM (%)	SEFEM Case 2	Error from SEFEM (%)
1	2.48	2.49	0.4	2.49	0	2.48	0.40
2	3.43	3.46	0.86	3.46	0	3.45	0.29
3	3.58	3.61	0.83	3.61	0	3.59	0.55
4	9.77	9.71	0.61	9.71	0	9.71	0
5	9.96	9.94	0.2	9.93	0.10	9.94	0
6	10.26	10.3	0.38	10.37	0.68	10.37	0.68
7	10.30	10.46	1.52	10.45	0.09	10.45	0.09
8	23.90	24.35	1.84	23.42	3.89	24.03	1.33
9	41.97	42.91	2.19	40.42	5.93	42.78	0.30
10	43.03	43.33	0.69	42.7	1.46	43.03	0.69
11	49.87	50.23	0.71	49.89	0.68	50.10	0.26
12	60.42	60.68	0.42	60.13	0.91	58.65	3.35
13	70.28	70.39	0.15	68.93	2.07	68.72	2.37
14	72.66	72.37	0.4	71.68	0.94	71.75	0.85
15	84.97	85.05	0.09	84.72	0.38	82.97	2.44
16	88.69	89.02	0.37	88.07	1.07	88.04	1.10
17	105.17	107.74	2.38	107.08	0.62	105.36	2.26

**Table 3:** CFRP Composite Beam Properties.

**Layer Orientation**	(+/−8°) (+86°) (+/−8°) (+/−8°)			
**Beam Internal Diameter**	25 mm		**Length**	1 m
**Layer Thickness**	+/−8°	0.52 mm		
	+86°	0.16 mm		

**Table 4:** Updated FE model material properties.

Part				1				
Parameter				Bounds			Result	
$\mathrm{Modulus}\;\mathrm{of}\;\mathrm{Elasticity},\;\mathrm{Direction}\;1,\;E_{1}\;[\mathrm{GPa}]$				[97, 150]			108.1	
$\mathrm{Modulus}\;\mathrm{of}\;\mathrm{Elasticity},\;\mathrm{Direction}\;2,\;E_{2}\;[\mathrm{GPa}]$				[4.6, 8.6]			8.061	
In-plane Shear Modulus $G_{12}\;[\mathrm{GPa}]$				[2.3, 4.4]			4.387	
Density, ρ $\left[{\mathrm{kg}/m}^{3}\right]$				[936, 2184]			1860	
**Part**	**2**		**3**		**4**		**5**	
**Parameter**	**Bounds**	**Result**	**Bounds**	**Result**	**Bounds**	**Result**	**Bounds**	**Result**
Young’s Modulus Ε [GPa]	[199, 220]	201.5	[199, 220]	208.8	[0.55, 1.7]	0.57	[61.0, 75.9]	62.55
Density ρ $\left[{\mathrm{kg}/m}^{3}\right]$	[7457, 8242]	7890	[7457, 8242]	7790	[490, 1474]	513	[2430, 2970]	2440

**Table 5:** Comparison of the Experimental natural frequencies with the Nominal (before the model update procedure) and Optimal Finite Element Model (after the model update procedure).

Mode	Experimental Freq. (Hz)	Nominal FEM Freq. (Hz)	Error (%)	Optimal FEM Freq. (Hz)	Error (%)
1	136.0	178.6	31.3	138.6	1.94
2	169.0	210.5	24.5	170.4	0.83
3	255.0	341.3	33.8	260.4	2.14
4	476.6	587.2	23.2	485.1	1.79
5	503.4	600.0	19.1	506.5	0.62

## Data Availability

Data set available on request to corresponding authors.
